# Microfiber Optical Sensors: A Review

**DOI:** 10.3390/s140405823

**Published:** 2014-03-25

**Authors:** Jingyi Lou, Yipei Wang, Limin Tong

**Affiliations:** 1 College of Science, Zhejiang University of Science and Technology, Hangzhou 310023; China; E-Mail: loujingyi@zust.edu.cn; 2 State Key Lab of Modern Optical Instrumentation, Zhejiang University, Hangzhou 310027; China; E-Mail: wyp154@zju.edu.cn

**Keywords:** optical microfiber, optical nanofiber, fiber taper, sensor

## Abstract

With diameter close to or below the wavelength of guided light and high index contrast between the fiber core and the surrounding, an optical microfiber shows a variety of interesting waveguiding properties, including widely tailorable optical confinement, evanescent fields and waveguide dispersion. Among various microfiber applications, optical sensing has been attracting increasing research interest due to its possibilities of realizing miniaturized fiber optic sensors with small footprint, high sensitivity, fast response, high flexibility and low optical power consumption. Here we review recent progress in microfiber optical sensors regarding their fabrication, waveguide properties and sensing applications. Typical microfiber-based sensing structures, including biconical tapers, optical gratings, circular cavities, Mach-Zehnder interferometers and functionally coated/doped microfibers, are summarized. Categorized by sensing structures, microfiber optical sensors for refractive index, concentration, temperature, humidity, strain and current measurement in gas or liquid environments are reviewed. Finally, we conclude with an outlook for challenges and opportunities of microfiber optical sensors.

## 1. Introduction

In the past 50 years, fiber-optical sensing has been one of the most successful and powerful applications of both fiber optics and sensing technology [1]. Recently, along with the rapid progress in micro/nanotechnology and increasing demands on optical sensors with higher performances and versatilities, spatial miniaturization has been one of the current trends of fiber-optic sensors. It is obvious that, reducing the size of a sensing structure is usually an essential step to bestow the sensor with faster response, higher sensitivity, low power consumption and better spatial resolution, and an optical microfiber is one of the best candidates for this purpose [2–4].

As a combination of fiber optics and nanotechnology, the optical microfiber (also called a nanofiber when its diameter is below 1 μm) has been emerging as a novel platform for exploring fiber-optic technology on the micro or nanoscale [2,5–8]. Fabricated by taper-drawing of glass or polymer materials (e.g., glass optical fibers), a microfiber usually has a diameter of hundreds of nanometers to several micrometers, excellent diameter uniformity and sidewall smoothness [9]. With high-index contrast between the microfiber material (e.g., glass or polymer) and the surrounding (e.g., air or water), this kind of micro or nanoscale waveguide guides light with low optical loss, outstanding mechanical flexibilities, tight optical confinement and large fractional evanescent fields [2,10], making it a novel miniaturized platform for optical sensing with special advantages including faster response, higher sensitivity, low power consumption.

Based on optical microfibers, a variety of physical, chemical or biological optical sensors have been demonstrated so far [2,5–8]. Here we review the recent progress in microfiber optical sensors regarding their waveguide properties, fabrication, and sensing applications. Typical microfiber-based sensing structures, including biconical tapers, optical gratings, circular cavities, Mach-Zehnder interferometers and functionally coated/doped microfibers, are summarized. Categorized by sensing structures, microfiber optical sensors for refractive index, concentration, temperature, humidity, strain and current measurement in gas or liquid environments are reviewed. Finally, we conclude with an outlook for challenges and opportunities of microfiber optical sensors.

## 2. Microfiber Optics for Sensors

**Figure 1.**
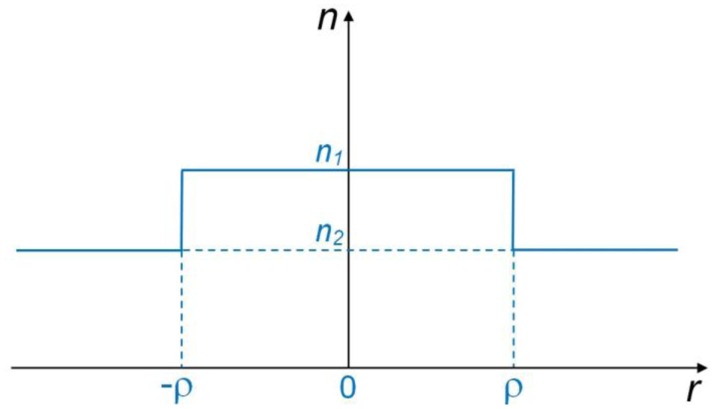
Index profile of an optical microfiber.

The basic model for microfiber optics is illustrated in Figure 1, in which the refractive indices of the microfiber and the surrounding are assumed to be *n*_1_ and *n*_2_, respectively. With the microfiber radius of ρ, the step-index profile of a waveguiding microfiber is then expressed as:

(1) $$n(r)=\{\begin{array}{c} \begin{array}{cc} n_{1}, & 0<r<\rho , \end{array}\\ \begin{array}{cc} n_{2}, & \rho \leq r<\infty \end{array} \end{array}$$

For non-absorptive materials, the waveguide parameters are determined by analytically solving the Helmholtz equations [11]:

(2) $$\begin{array}{c} (\nabla^{2}+n^{2}k^{2}-\beta^{2})\vec{e}=0\\ (\nabla^{2}+n^{2}k^{2}-\beta^{2})\vec{h}=0 \end{array}$$

where *k* = 2*π*/λ, λ is the wavelength of the light in vacuum, and β is the propagation constant.

With circular cross section, Equation (2) can be solved in cylindrical coordinate [10], with eigenvalue equations:

(3) $$HE_{vm}\mathrm{and}EH_{vm}\mathrm{modes}:\{\frac{J_{v}^{'}(U)}{UJ_{v}(U)}+\frac{K_{v}^{'}(W)}{WK_{v}(W)}\}\{\frac{J_{v}^{'}(U)}{UJ_{v}(U)}+\frac{{n_{2}}^{2}K_{v}^{'}(W)}{{n_{1}}^{2}WK_{v}(W)}\}={\left(\frac{\nu \beta }{kn_{1}}\right)}^{2}{\left(\frac{V}{UW}\right)}^{4}$$

(4) $$TE_{0m}\;\text{modes}:\frac{J_{1}(U)}{UJ_{0}(U)}+\frac{K_{1}(W)}{WK_{0}(W)}=0$$

(5) $$TM_{0m}\;\text{modes}:\frac{{n_{1}}^{2}J_{1}(U)}{UJ_{0}(U)}+\frac{{n_{2}}^{2}K_{1}(W)}{WK_{0}(W)}=0$$

where *J_v_* is the Bessel function of the first kind, and *K_v_* is the modified Bessel function of the second kind, and *U* = ρ(*k*_0_^2^*n*_1_^2^ – β^2^)^1/2^, *W* = ρ(β^2^ − *k*_0_^2^*n*_2_^2^)^1/2^, *V* = *k*_0_·ρ(*n*_1_^2^ − *n*_2_^2^)^1/2^.

By numerically solving Equations (2)–(5), the propagation constants of waveguiding modes (β) can be obtained. Generally, when its diameter goes close to or smaller than the wavelength of the guided light, a microfiber with a much-lower-index surrounding (e.g., air or water) offers favorable properties for optical sensing, including tight optical confinement, high fractional evanescent fields and low bending loss.

**Figure 2.**
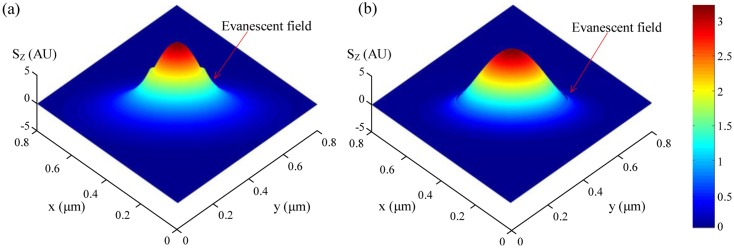
*Z*-Components of the Poynting vectors (*S_z_*) of the *HE*_11_ mode of a (**a**) 200 and (**b**) 400 nm diameter silica microfibers at 325 nm wavelength [12].

**Figure 3.**
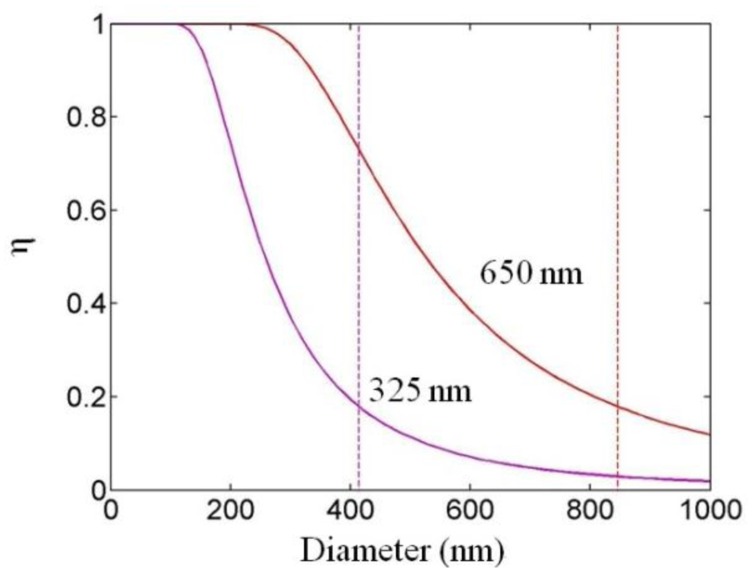
Fractional power of the fundamental mode outside the core of silica microfibers at 325 and 650 nm wavelength. Dashed lines: single-mode cutoff diameters [12].

For optical sensing applications the microfiber is usually operated in single mode, and it is important to know the fractional power of optical fields guided outside the microfiber. Using propagation constants obtained by numerically solving Equation (2), the profile of the evanescent fields and the power distribution around the microfiber can be obtained. For example, Figure 2 shows the *z*-components (the only non-zero component along the axial direction of the microfiber) of the Poynting vectors (*S_z_*) of the *HE*_11_ mode of a 200 and 400 nm diameter silica microfiber in water at 325 nm wavelength [12], respectively. The refractive indices of the silica (1.457 at 650 nm wavelength, and 1.482 at 325 nm wavelength) and water (1.333 at 650 nm wavelength, and 1.355 at 325 nm wavelength) are obtained from their dispersion formulas at room temperature [13,14]. The evanescent field (separated from the central peak with a discontinuous gap) outside the guiding microfiber core is clearly seen, which is very sensitive to index change of the surrounding media. Compared with the 400 nm diameter one, the 200 nm diameter microfiber offers much higher fraction of evanescent waves, and thus much more sensitive to the surroundings. Calculated fractional power of the evanescent wave outside the microfiber at 325 and 650 nm wavelengths is also shown in Figure 3 [12]. Depends on the diameter, a single-mode silica microfiber can guide light with about 20 to almost 100 percent of energy as evanescent waves, which has been confirmed by previous reports [9,15,16] and is difficult to achieve in many other optical waveguides with much higher sidewall roughness. The high fractional evanescent fields is very helpful for achieving high sensitivity for index measurement in homogeneously distributed samples [4]. Moreover, benefitted from the large index contrast, a microfiber can offer high fractional evanescent fields with tight spatial confinement.

**Figure 4.**
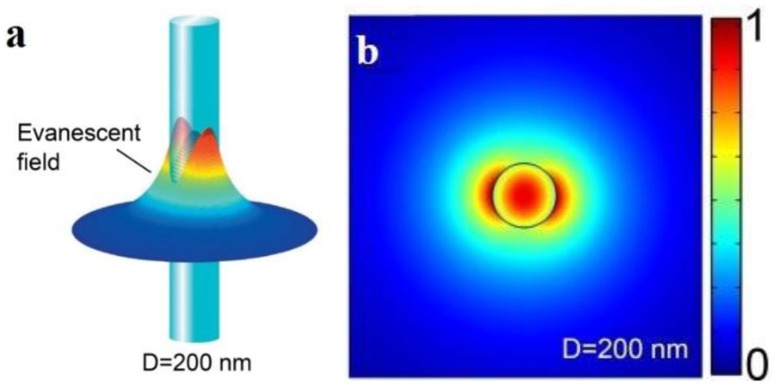
*Z*-direction Poynting vectors of a 400 nm diameter silica microfiber at 633 nm wavelength (**a**) in 3-D view; and (**b**) in 2-D view [3].

For example, Figure 4 shows the *z*-components of the Poynting vectors (*S_z_*) of the *HE_11_* mode of a 200 nm diameter silica microfiber guiding a 633 nm light in air, with a fractional power of about 90% guided as evanescent fields outside the microfiber, the guiding mode is confined within a 600 nm area [3]. The tightly confined high fractional evanescent fields is especially desired for ultra-sensitive optical sensing of localized samples in forms of micro or nanoparticles [4,12,17].

## 3. Microfiber Fabrication

Typical microfibers are fabricated from amorphous materials such as glass and polymers. For these amorphous materials, the top-down physical drawing is proved very successful for yielding microfibers with circular cross-section, uniform diameter, smooth surface and large length [2]. Here we briefly introduce the typical fabrication techniques for glass and polymer microfibers, detailed fabrication techniques have been reviewed somewhere else [2,7].

**Figure 5.**
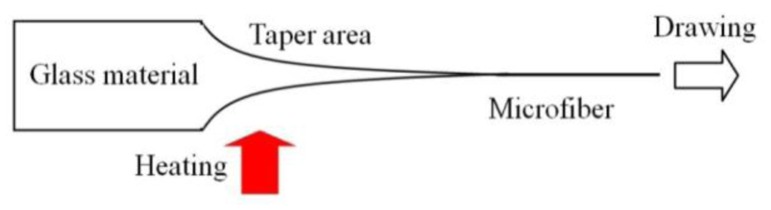
Schematic diagram of taper-drawing technique.

Glass microfibers are usually fabricated by taper drawing of glass fiber or bulk glass at high temperature, as illustrated in Figure 5. The heating source can be a flame (usually a hydrogen flame) [9,15,18–21], an electrical heater [22–24] or a laser-heated tube [25–28]. Depending on the heating conditions and the material properties around the melting temperature, the drawing speed varies from tens of micrometers to several meters per second. By precisely controlling the drawing speed, heating conditions and subsequently the geometric profile of the tapering region, an microfiber drawn from a standard glass fiber, with both ends naturally connected to the original fiber (usually called a biconical microfiber or fiber taper), can exhibit high- efficiency (up to almost 100%) or so-called adiabatic connection to the optical fiber, making it very convenient for in/out-coupling of light via standard optical fiber system.

**Figure 6.**
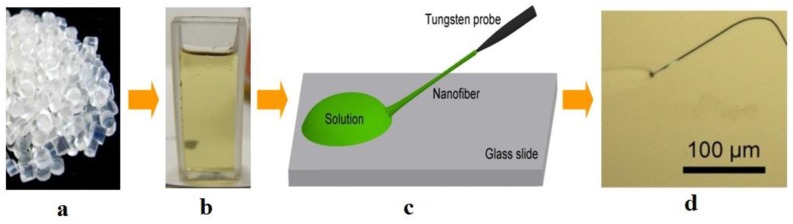
Illustration of direct drawing a polymer microfiber from a solution. Bulk polymer materials (**a**) is firstly dissolved in a certain solvent (**b**), and a droplet of polymer solution is picked up and placed upon a substrate and drawn by a tip after evaporation of the solvent to a certain degree (**c**), then the tip is withdrawn with a speed of 0.1–1 m/s to form a polymer microfiber (**d**).

Polymer microfibers are usually fabricated by direct drawing of polymer solutions at room temperature. As shown in Figure 6, polymer bulk materials is firstly dissolved in a certain solvent to form a homogeneous polymer solution, and a droplet of polymer solution is picked up and placed upon a substrate (e.g., a glass slide) by a certain tip (e.g., tungsten probe); with the evaporation of the solvent, the viscosity of the solution gradually increases to an appropriate value for drawing; then, the tip is withdrawn with a speed of 0.1–1 m/s, and a polymer microfiber can be formed with excellent geometric uniformity.

**Figure 7.**
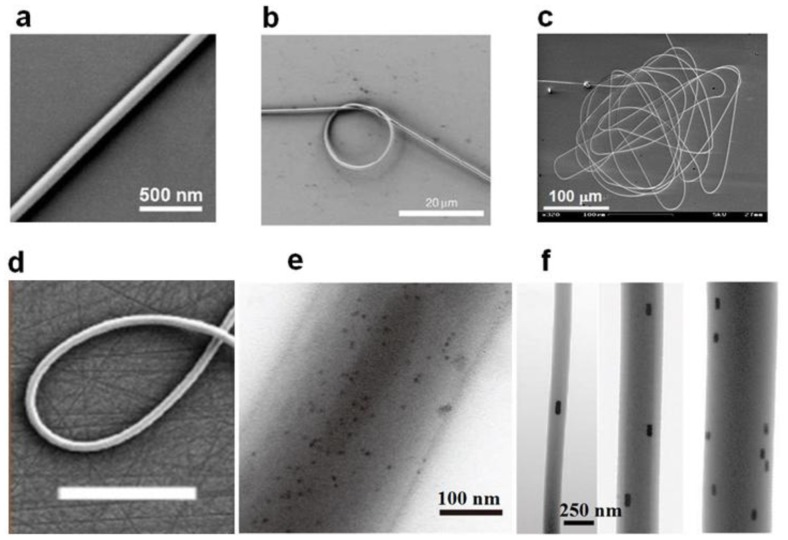
(**a**) SEM image of a 100 nm diameter tellurite glass microfiber [29]; (**b**) SEM image of a 15 mm diameter micro-ring made with a 520 nm diameter silica microfiber [9]; (**c**) A coiled 260 nm diameter silica microfiber with a total length of about 4 mm [9]; (**d**) SEM image of a 350 nm dameter PMMA microfiber. Scale bar: 50 μm; (**e**) A 280 nm diameter polystyrene (PS) microfiber doped with CdSe quantum dots [30]; (**f**) PAM microfiber doped with aligned GNRs [31].

Typical electron microscope images of physically drawn glass and polymer microfibers are shown in Figure 7.

## 4. Microfiber Functionalized Structures and Optical Sensors

Depends on the sensing mechanism and the sample to be measured, so far, a number of microfiber-based functional structures have been demonstrated. Here we summarize the typical functional structures including biconical microfibers, microfiber gratings, microfiber circular cavities, microfiber Mach-Zehnder interferometers (MZIs), and some other microfiber-based structures, the corresponding sensors, and the advantages and/or potentials of these miniaturized optical sensors.

### 4.1. Biconical Microfibers

**Figure 8.**
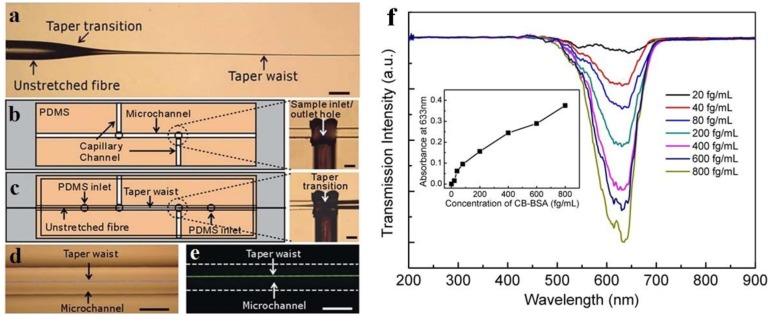

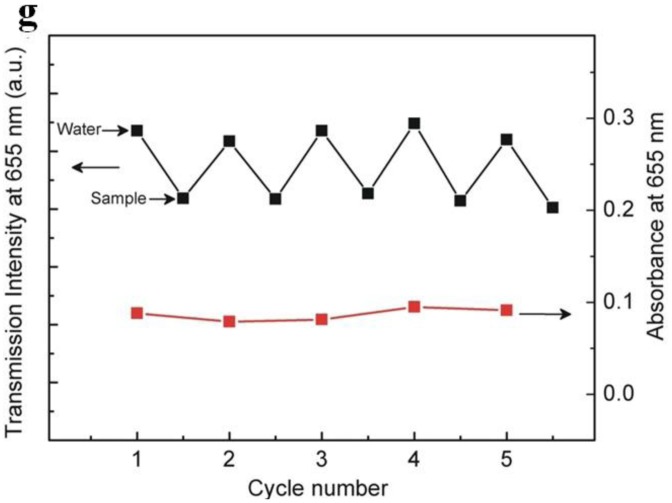
Microfiber absorption sensor [33]. (**a**) Biconical tapered fiber with a 900 nm diameter waist (microfiber). Scale bars: 125 μm; (**b**,**c**) Cartoon and optical micrographs of microfluidic chip based microfiber sensor fabrication procedures; (**d**) Optical micrograph of a 1.5 μm diameter microfiber guiding a laser with a wavelength of 473 nm embedded in a microchannel; (**e**) Optical micrograph of the fluorescence excited by evanescent field outside a 1.5 μm diameter microfiber. Scale bars: 125 μm; (**f**) Transmission spectra of different MB concentrations for the sensor with a 900 nm diameter microfiber. Inset: absorbance at 635 nm wavelength *versus* MB concentrations for two microfibers with diameters of 900 nm and 1.5 mm, respectively; (**g**) Cycling measurement with 500 pM MB solutions for a 900 nm diameter microfiber. The *y*-errors are determined from three repeated measures.

One of the most straightforward approaches to a microfiber optical sensor is using a biconical microfiber or fiber taper, which has been successfully applied in measuring a variety of samples. Due to its simple configuration, this kind of sensor usually measures the intensity transmission of the microfiber, and retrieve the sample information by concentration-dependent optical intensity. In 2007, relying on absorption of molecules adsorbed on the surface of a 500 nm diameter microfiber, Warken *et al.* reported an ultra-sensitive molecular sensor that was possible to detect sub-monolayers of 3,4,9,10-perylenetetracarboxylic dianhydride (PTCDA) molecules at ambient conditions [17]. Compared to microfiber sensors for air or gas that usually has a refractive index close to unit, the microfiber used for liquid (that has an index much higher than the air) sensing usually has a larger diameter. In 2005, Polynkin *et al.* reported a microfiber optical sensor for measuring the refractive index of liquids in microfluidic channels [32]. By measuring the refractive-index-dependent leakage loss of 1.5 μm wavelength light guided in a 700 nm diameter silica microfiber, they realized an accuracy of refractive-index measurement of 5.3 × 10^−4^. In 2011, by integrating a 900 nm diameter microfiber into a 125 μm wide microfluidic channel, Zhang *et al.* demonstrated a microfiber sensor for chemical and biological applications (Figure 8) [33]. As shown in Figure 8f, using a broadband white light as probing light with power of about 150 nW, the absorbance of bovine serum albumin (BSA) was clearly observed with a detection limit down to 10 fg·mL^−1^. In addition, Figure 8f provides transmission intensity of the microfiber response to analytes cycled with 500 pM methylene blue (MB) solution and ultrapure water, showing good absorbance reversibility of the sensor.

### 4.2. Microfiber Gratings

Like optical fiber gratings [2,34], microfiber gratings have been attracting increasing interest for optical sensing in recent years [8]. Owing to their high-compactness, strong near-field interaction with the surrounding material, and high-resistance to mechanical and thermal shocks, microfiber gratings offer special advantages for sensing including high sensitivity, small footprint, large dynamic range, low detection limit and fast response. According to their structures, microfiber gratings can be categorized into three types: Bragg gratings, long period gratings, and evanescently coupled gratings. So far, a variety of techniques has been reported for fabrication of microfiber gratings [35–44], which has been reviewed elsewhere [3,8]. Here we introduce optical sensors relying on the three types of microfiber gratings mentioned above.

#### 4.2.1. Microfiber Bragg Gratings

**Figure 9.**
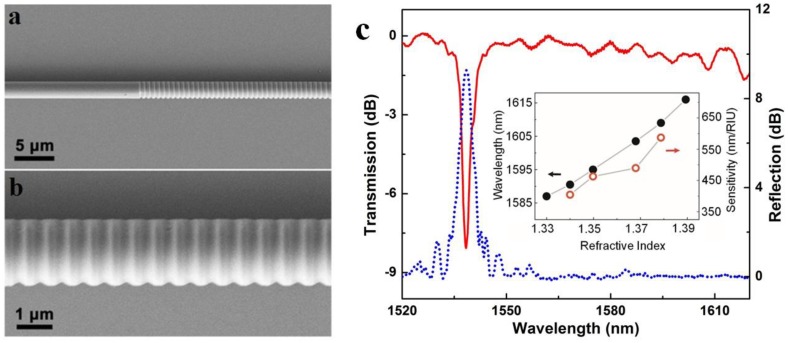
microfiber Bragg gratings (MFBG) [42]. (**a**) Scanning electron microscope (SEM) image of a MFBG inscribed on a 1.8 μm diameter silica microfiber; (**b**) Close-up view of the MFBG; (**c**) Transmission and reflection spectra of the MFBG. Inset is dependence of the reflection wavelength shift on the ambient RI (black dot line) and the corresponding RI sensitivity (red hollow dot line) of the MFBG used for measuring the RI of a glycerin solution.

In principle, a microfiber Bragg gratings (MFBG) is a miniaturized copy of a standard fiber Bragg gratings (FBG), which spectrally manipulate waveguided light by periodically modified refractive index along the length of the fiber. However, due to its much smaller diameter and much more compact overall size, to obtain an evident grating effect, the index-contrast of the microfiber grating is usually much higher. In 2005, Liang *et al.* fabricated an MFBG by chemically etching a 6 μm diameter silica microfiber, which was successfully used for optical index sensing in liquids [39]. In 2010, Fang *et al.* reported MFBGs fabricated by femtosecond laser pulse irradiation. Using a 2 μm diameter silica microfiber gratings, they reported a maximum sensitivity of 231.4 nm/RIU at a refractive index (RI) value of 1.44 for RI measurements [38]. Around the same time, Zhang *et al.* reported a MFBG written in a photosensitive microfiber using KrF excimer laser, and demonstrated a sensitivity of 102 nm/RIU (at a RI value of 1.378) in a 6 μm diameter microfiber [36]. Shortly after, using focused ion beam to milling the sidewall of a microfiber, Liu *et al.* demonstrated a 518 μm length 1.8 μm diameter MFBG and a sensitivity of 660 nm/RIU for RI measurement around 1550 nm wavelength (Figure 9) [42]. Around the same time, Kou *et al.* reported a MFBG fabricated by FIB milling, and demonstrated temperature sensing from room temperature to around 500 °C with a sensitivity of nearly 20 pm/°C near the resonant wavelength of 1550 nm [41]. In addition, with similar other parameters, MFBG with thinner fiber and higher order mode can result in higher RI sensitivity [37].

#### 4.2.2. Long period Gratings

Microfiber long period grating (LPG) has also been reported for optical sensing. Using a femtosecond IR laser to periodically modify the surface of wavelength-diameter (1.5–3 μm) microfiber, Xuan *et al.* reported a microfiber LPG with periods of 10–20 μm, which exhibited strong resonant dip as high as 22 dB around 1,330 nm with only 10 periods [35]. Later in 2010, a 20-period LPG with a 27 dB attenuation dip was further demonstrated in a 6.3 μm diameter microfiber, with sensitivity of −130 pm/°C and 1,900 nm/RIU for temperature and refractive index sensing, respectively [45].

#### 4.2.3. Evanescently Coupled Gratings

Evanescently coupled gratings are manufactured by wrapping a microfiber on a micro-structured rod [46]. The rod is designed with an inner hollow channel for microfluidic sensing and some air-holes arranged in the outer circle. By exploiting the large evanescent field in an inner channel, microfluidic refractometric sensors based on the evanescently coupled gratings can be achieved with a sensitivity higher than 10^3^ nm/RIU [47].

### 4.3. Microfiber Circular Cavities

**Figure 10.**
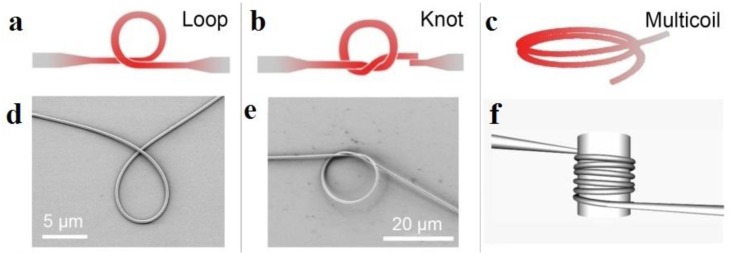
Optical microfiber resonators. Schematics of (**a**) loop; (**b**) knot; (**c**) multicoil resonators [3]; (**d**) SEM image of a loop resonator; (**e**) SEM image of knot resonator using a 520 nm diameter silica microfiber [9]; (**f**) Schematic of an optical-rod-wrapped microfiber multicoil resonator [48].

When a microfiber is assembled into a closed loop, a microfiber-based circular cavity can be formed via evanescent coupling at the overlapping area. Depends on the geometric parameters and the coupling condition, the Q-factor of a microfiber cavity varies from several hundreds to higher than one million [2]. According to their structures, so far there're four kinds of microfiber circular cavities: loop, knot and multi-coil, as shown in Figure 10.

#### 4.3.1. Microfiber Loops

Among microfiber cavities, the microfiber loop has the simplest structure, and has been intensively investigated in the past years. In 2006, Sumetsky *et al.* reported an microfiber loop cavity with a loaded Q-factor of 120,000 and an intrinsic *Q*-factor of 630,000, and successfully used the micro-cavity as an ultrafast direct contact temperature sensor [49]. Benefitted from the high Q-factor and the miniaturized structure, the temperature resolution can be as small as ∼0.1 mK, with a response time down to microsecond level.

**Figure 11.**
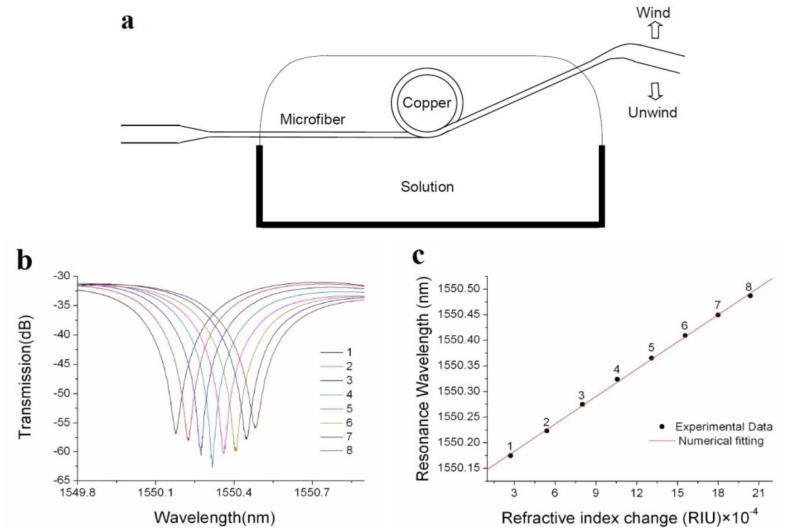
Refractive-index sensor based on copper-rod-supported microfiber loops [51]. (**a**) Schematic side view of a copper-rod-supported microfiber loop; (**b**) Spectral shifts of a resonant peak caused by index change of the solution. The eight peaks are obtained by adding a 5 μL ethanol into a 500 μL water in steps. The loop is about 480 μm in diameter and is assembled with a 2.4 μm diameter microfiber; (**c**) Resonant wavelength as a function of the refractive index change. The black dots are resonant wavelengths extracted from (**b**), and the numerical fitting is obtained with a calculated slope of 17.8 (nm/RIU).

Although the free-standing microfiber loop is easy to fabricate, since the loop structure is maintained by van der Vaals or electrostatic forces at the joint area, it is mechanically fragile, especially in liquid environment. To enhance the robustness of a microfiber loop for operating in liquid environment, in 2007 Guo *et al.* reported an copper-rod-supported loop resonator assembled by wrapping a 2.8 μm diameter microfiber around a 460 μm diameter copper rod [50], with a maximum extinction of 30 dB and a *Q* of about 4000 around 1.53 μm wavelength. With the copper-rod supporting, the microfiber loop exhibits high stability and flexibility of achieving critical coupling within a broad spectral range [51], enabling high-sensitivity optical sensing in both low- and high-concentration solutions (Figure 11), with estimated sensitivity of refractive-index measurement up to 1.8 × 10^−5^.

Besides the above-mentioned schemes, embedding a free-standing microfiber loop inside a low-index substrate (e.g., a polymer matrix) is another possible route to fabricate a robust micro-resonator for sensing applications [52–54].

#### 4.3.2. Microfiber Knots

**Figure 12.**
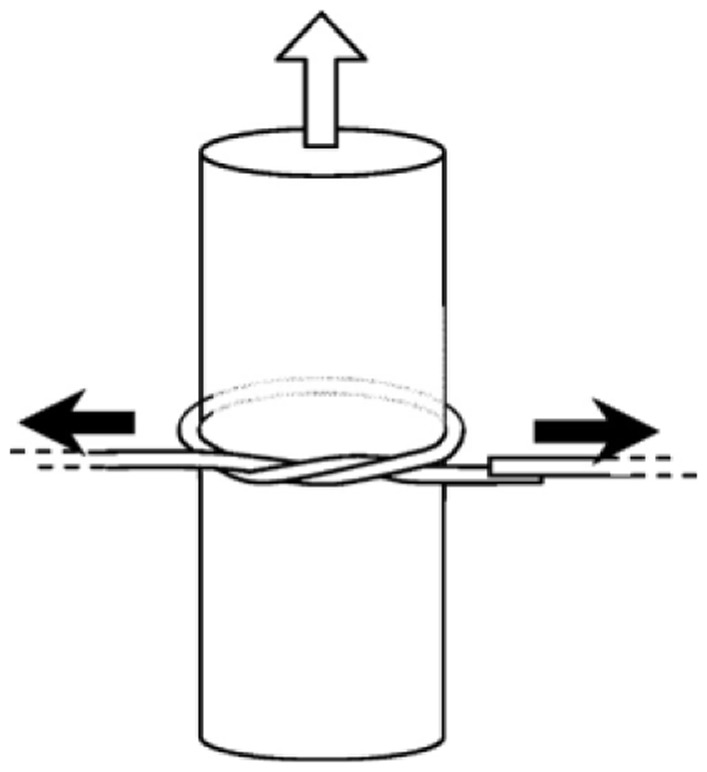
Schematic illustration of microfiber knot resonator tied on a copper rod. The upward arrow in the figure indicates the direction of the electric current in the copper rod [68].

Supporting a microfiber loop with a certain substrate has been proved to be an effective approach to microfiber-loop sensors with higher robustness, however, the additional substrate inevitably increases the complexity and overall size of the sensing element. In 2006, Jiang *et al.* proposed to tie a free-standing microfiber into a knot [55], in which the knot structure was maintained by the friction of the microfiber at the joint area under the tension of the elastically bent knot, and was proved highly stable in water with *Q* factors up to 31,000 and finesse of 13. Based on microfiber knot structures, a variety of sensing applications have been reported [56–66]. In 2009, Wu *et al.* demonstrated a microelectromechanical system (MEMS) based optical accelerometer combined with a 386 μm diameter microfiber knot resonator fabricated by a 1.1 μm diameter silica microfiber [67]. The microfiber knot had a *Q*-factor of 8500 and was used for vibration measurement of the MEMS structure. The experimental results showed that the microfiber accelerometer had a sensitivity of 654.7 mV/g, with a dynamic range of 20 g. In 2011, using a copper-wire-wrapped microfiber knot resonator (Figure 12), Lim *et al.* demonstrated tuning the resonant wavelength of the microfiber resonator by applying electric current to the copper wire [68], and realized a compact current sensor with the maximum tuning slope of 51.3 pm/A^2^. Besides the above-mentioned examples, microfiber-knot-based optical sensors have also been reported for measurement of refractive index [64,69], humidity [61,70], temperature [57,69,71,72], and magnetic field [73]. Overall, as a free-standing microfiber cavity without the supporting substrate, the microfiber knot resonator offers opportunities for optical sensing with high sensitivity, fast response, high robustness and compact size in liquid and/or vibrating environment.

#### 4.3.3. Microfiber Multicoil Resonator (MMR)

Firstly proposed by Sumetsky in 2004 [26], the three-dimensional multicoil resonators are usually fabricated by wrapping a microfiber around a low-index rod in several turns, as shown in Figures 10c,f. In 2007, Xu *et al.* calculated a refractometric sensor based on a coated MMR, and predicted a sensitivity up to 700 nm/RIU [74]. Shortly after, the same group experimentally demonstrated [75] a refractometric sensor based on a 5-turn Teflon-coated 3D MMR that is assembled with a 50 mm length 2.5 μm diameter silica microfiber. The sensor was operated in a fluidic channel, and offered a sensitivity of about 40 nm/RIU for index sensing of mixtures of isopropyl of methanol. Since then, a number of MMR optical sensors have been reported for optical sensing of absorption [76], acoustic waves [77], and temperature [78]. Theoretically, an MMR can offer *Q*-factor up to 10^10^ [49], which may lead to very high optical sensitivity. However, due to the complicated structure and high precision requirement, experimental *Q*-factor is limited, leaving a lot of space for future improvement.

### 4.4. Microfiber MZIs

**Figure 13.**
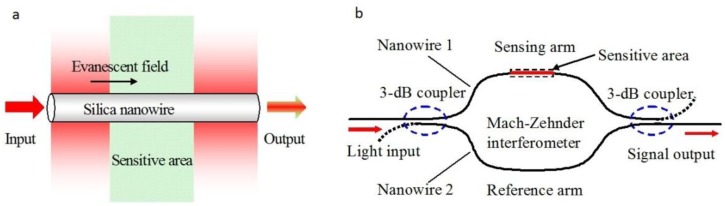
Schematic diagram of (**a**) the silica microfiber sensing element; and (**b**) the proposed sensor with a Mach-Zehnder interferometer [12].

Optical interferometers are among the most powerful structures for optical sensing. As a miniaturized optical interferometer, the microfiber MZI is of particular interest for phase-sensitive optical measurement with compact size and high sensitivity. In 2005, based numerical calculation, Lou *et al.* modeled a microfiber-MZI optical sensor (Figure 13) for refractive index measurement in liquid environment, and predicted a sensitivity one order of magnitude higher than those of conventional waveguide MZIs [12].

**Figure 14.**
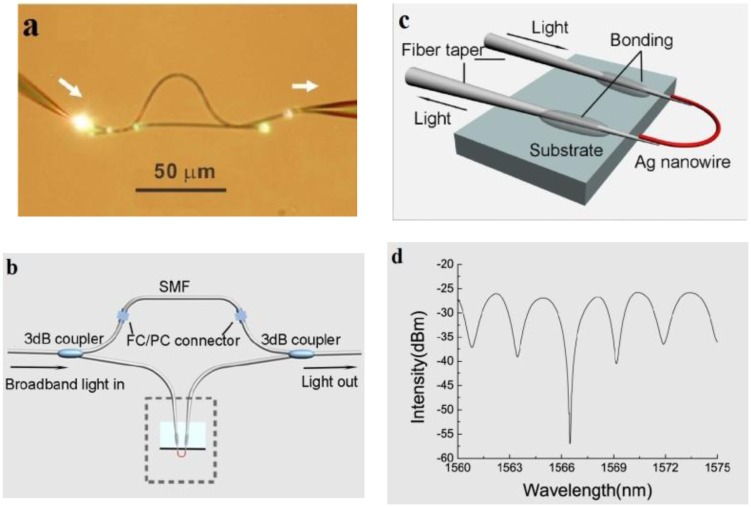
Microfiber-based MZI. (**a**) Optical microscope image of a MZI assembled with two 480 nm diameter tellurite microfibers. White light from a supercontinuum source is launched into and picked up from the MZI by two silica fiber tapers. The white arrows indicate the direction of light propagation [79]; (**b**) Schematic of a hybrid photonic-plasmonic MZI. The structure in the dashed box represents the in-fiber return-signal plasmonic probe; (**c**) A closed-up view of the plasmonic probe; (**d**) Typical transmission spectrum of the hybrid MZI [82].

**Figure 15.**
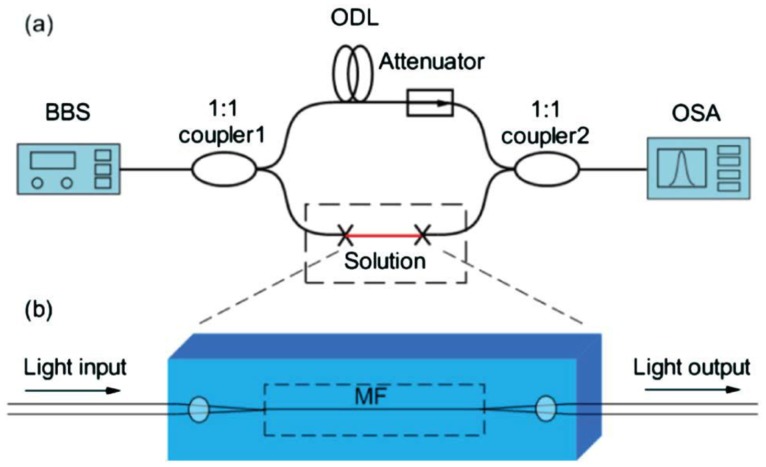
(**a**) Schematic configuration of the MZI-based RI sensor; (**b**) Schematic diagram of the sensing arm. BBS, broadband light source; ODL, optical delay line; OSA, optical spectrum analyzer; MF, microfiber [80].

In 2008, Li *et al.* experimentally demonstrated microfiber MZIs assembled from silica and tellurite glass microfibers, with microfiber diameter down to 480 nm and footprints down to 50 μm (Figure 14a) [79]. In 2012, Wo *et al.* reported a simple and robust refractive index sensor based on a microfiber MZI, as shown in Figure 15 [80]. Using a 2 μm diameter microfiber as the sensing arm, the sensor realized a RI sensitivity of 7159 μm/RIU. Jasim *et al.* reported a microfiber-MZI-based current sensor, with a slope efficiency of 60.17 pm/A^2^ [81]. Very recently, by integrating a Ag nanowire with a microfiber MZI (Figure 14b–d), Li *et al.* demonstrated a hybrid photon-plasmon MZI for fiber-compatible plasmonic sensing, with a response time of 0.3 s and a sensitivity better than 100 ppm for NH_3_ gas sensing [82].

### 4.5. Functionally Activated Microfibers

**Figure 16.**
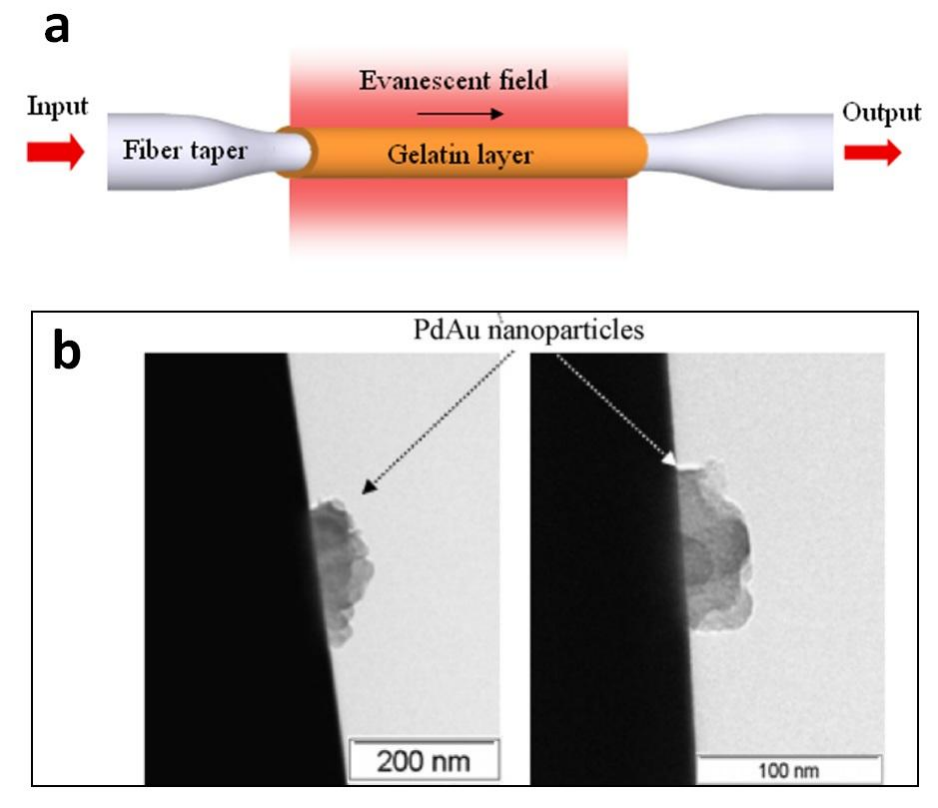
Functionally activated silica microfibers. (**a**) Schematic illustration of a gelatin coated microfiber for RH sensing [84]; (**b**) TEM images of microfibers (black cylinder) decorated with PdAu nanoparticles for hydrogen sensing [85].

**Figure 17.**
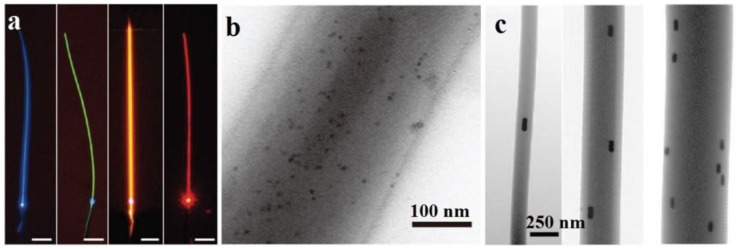
Functionally activated polymer microfibers. (**a**) Typical light-emitting polymer microfibers excited by 355 nm light. The microfibers are doped with different fluorescent dyes to emit different colors of light. Scale bars: 50μm [90]; (**b**) A 280 nm diameter PS microfiber doped with CdSe quantum dots [30]; (**c**) Three PAM nanofibers doped with aligned GNRs [31].

Typically, glass or polymer microfibers directly drawn from undoped materials are passive structures. Functional activation is desired for many sensing applications. For glass microfibers, one of the most convenient approaches is coating the microfiber with a functional film while maintaining its waveguiding capability. In 2005, based on a 1.3 μm diameter silica microfiber coated with an ultra-thin palladium film, Villatoro *et al.* reported a miniature hydrogen sensor operated at 1550 nm wavelength [83]. Relying on the hydrogen-concentration-dependent transmission intensity of the microfiber, they successfully demonstrated a fast-response (∼10 s) hydrogen sensor with low detection limit. In 2008, by coating a 80 nm thickness gelatin layer on the sidewall of a 680 nm diameter biconical microfiber (Figure 16), Zhang *et al.* reported a microfiber optical sensor operated at 1550 nm wavelength for fast detection of relative humidity (RH) [84]. When exposed to moisture, the change in refractive index of the gelatin layer changes the mode field of the guided mode, and converts a portion of power from guided mode to radiation mode, resulting in RH-dependent loss for optical sensing. The sensor is operated within a wide humidity range (9%–94% RH) with high sensitivity and good reversibility. Measured response time is about 70 ms, which is one or two orders of magnitude faster than other types of RH sensors relying on conventional optical fibers or films. In 2010, by decorating a 10 μm diameter silica microfiber with PdAu nanoparticles (Figure 17), Monzon-Hernandez *et al.* demonstrated a microfiber hydrogen sensor [85]. Relying on reversible optical transmission changes originated from hydrogen-concentration-dependent nanoparticle scattering, the sensor was capable of detecting low concentrations of hydrogen (up to 8%) at room temperature with response and recovery times on the order of seconds, which was faster than many other hydrogen sensors that exploit phenomena at the nanoscale. Using similar configurations, more functionally coated or decorated microfiber optical sensors have been reported recently [86,87].

**Figure 18.**
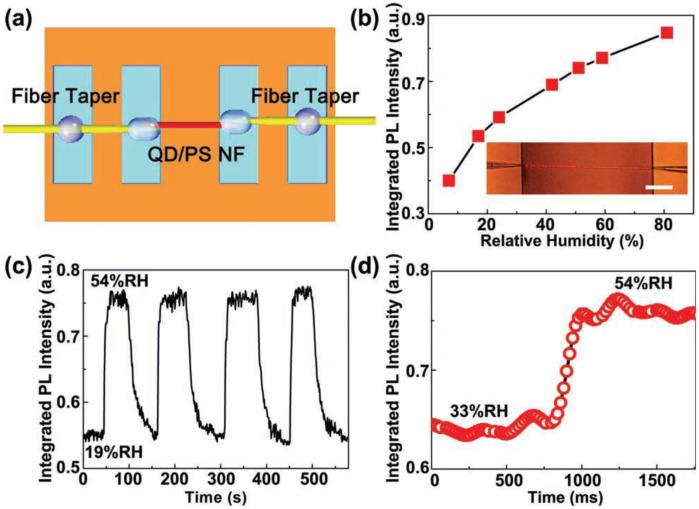
QD-doped microfiber optical sensor [30]. (**a**) Schematic illustration of a QD-doped PS microfiber optical sensor; (**b**) PL intensity of the microfiber exposed to ambient relative humidity (RH) ranging from 7% to 81%. Inset, optical microscopy image of the QD-doped microfiber sensing element. Scale bar: 50 μm; (**c**) Response of the MF sensor to alternately cycled 54% and 19% RH air; (**d**) Typical time-dependent integrated PL intensity of the microfiber reveals a response time of about 90 ms when RH jumps from 33% to 54%.

For polymer microfibers, owing to the excellent hospitality for exotic dopants, a number of functional dopants, including dye molecules [88–91], chemical indicators [92], quantum dots [30,93–95] and noble metal nanoparticles [31], have been doped inside waveguiding polymer microfibers for optical sensing (Figure 17). In 2008, based on spectral response of an acidic-to-basic form change of bromothymol blue (BTB) doped in a 270 nm diameter poly(methyl methacrylate) (PMMA) microfiber, Gu *et al.* demonstrated a microfiber NH_3_ sensor with a detection limit of 3 ppm and a response time of about 1.8 s, much faster than conventional ammonia sensors [92]. In 2010, to activate the microfiber with high resistance to photobleaching, Meng *et al.* doped a 480 nm diameter PS microfiber with CdSe/ZnS QDs, and realize a miniaturized optical humidity sensor based on the surface passivation of QD emission (Figure 18) [30]. The sensor offered a response time of 90 ms under a pumping power (532 nm CW light) of merely 0.1 nW, representing an ultra-low power optical nanosensor. More recently, by doping a 540 nm diameter polyacrylamide (PAM) microfiber with gold nanorods (GNRs) whose plasmonic resonance bands were highly dependent on environmental refractive index, Wang *et al.* demonstrated a low-power fast-response optical humidity sensor that is intrinsically immune to photobleaching [31].

### 4.6. More Microfiber Structures

Besides the above-mentioned microfiber structures, more microfiber-based structures, including twisted microfibers [96] and complex structures combining two or more microfiber structures have also been reported [61]. For example, in 2011, by twisting a pair of silica microfibers into a coupler, Liao *et al.* reported a simple and compact optical sensor for refractive index measurement in isopropanol/water solution [96]. By measuring the spectral shift of the modulated dip of the coupler, they obtained a maximum sensitivity up to 2377 nm/RIU at the RI value of 1.3680 with microfiber diameter of 2.8 μm, and the sensitivity can be further increased by decreasing the microfiber diameter. These versatile microfiber structures have provided more opportunities for microfiber optical sensors.

## 5. Conclusions and Outlook

So far, a variety of microfiber optical sensors have been proposed or experimentally demonstrated. Benefitting from their small sizes and high-fractional evanescent fields, microfiber sensors have shown special advantages (such as high sensitivity for refractive index measurement and fast response for temperature and humidity sensing) over conventional optical fiber sensors. Also, the tight confinement and surface enhancement of probing light waveguided along a microfiber, are beneficial for achieving high-sensitivity with extremely low optical power, which is highly desired for many applications.

As a future outlook, there are a number of opportunities and challenges for microfiber optical sensing, including (1) higher sensitivity: for example, single-molecule detection for bio-chemical optical sensing, <10 ppb detection limit for gas sensing, and <10^−6^ for refractive index sensing; (2) better selectivity: enabling selective detection of target samples by properly functionalizing the microfiber structure; (3) long-term stability: as a tiny structure highly sensitive to environmental changes (e.g., temperature and displacement), better robustness is highly desired for long-term use; (4) finally, better protection or package for practical applications.
